# Litter quality modulates changes in bacterial and fungal communities during the gut transit of earthworm species of different ecological groups

**DOI:** 10.1093/ismeco/ycae171

**Published:** 2024-12-26

**Authors:** Huizhen Chao, Linlin Zhong, Ina Schaefer, Mingming Sun, André Junggebauer, Feng Hu, Stefan Scheu

**Affiliations:** J.F. Blumenbach Institute of Zoology and Anthropology, University of Göttingen, Untere Karspüle 2, 37073 Göttingen, Germany; Soil Ecology Lab, Jiangsu Provincial Key Laboratory of Coastal Saline Soil Resources Utilization and Ecological Conservation, Jiangsu Collaborative Innovation Center for Solid Organic Waste Resource Utilization & Jiangsu Key Laboratory for Solid Organic Waste Utilization, Nanjing Agricultural University, Nanjing 210095, China; J.F. Blumenbach Institute of Zoology and Anthropology, University of Göttingen, Untere Karspüle 2, 37073 Göttingen, Germany; J.F. Blumenbach Institute of Zoology and Anthropology, University of Göttingen, Untere Karspüle 2, 37073 Göttingen, Germany; Loewe Center for Translational Biodiversity Genomics (LOEWE-TBG), 60325 Frankfurt Main, Germany; Senckenberg Biodiversity Climate Research Center, 60325 Frankfurt Main, Germany; Soil Ecology Lab, Jiangsu Provincial Key Laboratory of Coastal Saline Soil Resources Utilization and Ecological Conservation, Jiangsu Collaborative Innovation Center for Solid Organic Waste Resource Utilization & Jiangsu Key Laboratory for Solid Organic Waste Utilization, Nanjing Agricultural University, Nanjing 210095, China; J.F. Blumenbach Institute of Zoology and Anthropology, University of Göttingen, Untere Karspüle 2, 37073 Göttingen, Germany; Soil Ecology Lab, Jiangsu Provincial Key Laboratory of Coastal Saline Soil Resources Utilization and Ecological Conservation, Jiangsu Collaborative Innovation Center for Solid Organic Waste Resource Utilization & Jiangsu Key Laboratory for Solid Organic Waste Utilization, Nanjing Agricultural University, Nanjing 210095, China; J.F. Blumenbach Institute of Zoology and Anthropology, University of Göttingen, Untere Karspüle 2, 37073 Göttingen, Germany; Centre of Biodiversity and Sustainable Land Use, University of Göttingen, Büsgenweg 1, 37077 Göttingen, Germany

**Keywords:** earthworm ecological groups, gut passage, intestinal microbes, microbial diversity, NGS sequencing, resources quality

## Abstract

Earthworms are keystone animals stimulating litter decomposition and nutrient cycling. However, earthworms comprise diverse species which live in different soil layers and consume different types of food. Microorganisms in the gut of earthworms are likely to contribute significantly to their ability to digest organic matter, but this may vary among earthworm species. Here, we analyse the effect of food (litter) quality on gut microbiota and their changes during the gut passage (from foregut to hindgut) of earthworms of different ecological groups. The endogeic (soil living) species *Aporrectodea caliginosa* and the anecic (litter feeding) species *Lumbricus terrestris* were fed with high- (rape leaves) and low-quality litter (wheat straw) in a microcosm experiment for 18 weeks. Irrespective of earthworm species, alpha diversity of bacterial and fungal communities changed little during the gut passage, with the composition and diversity of microbial communities in the gut generally resembling those in soil more than in litter. In addition, the low-quality litter supported higher alpha diversity and more complex communities than high-quality litter. Further, gut microbial communities of the anecic *L. terrestris* changed less during gut passage than those of the endogeic *A. caliginosa*, especially when fed low-quality litter. Our findings indicate that earthworm gut microbial communities are predominantly shaped by the soil they ingest, but are modulated by the quality of litter they feed on and earthworm ecological group. Overall, the results suggest that earthworms primarily influence soil microbiota by mixing and spreading microorganisms from different microhabitats through bioturbation rather than by digesting microorganisms.

## Graphical Abstract

**Graphical Abstract:**
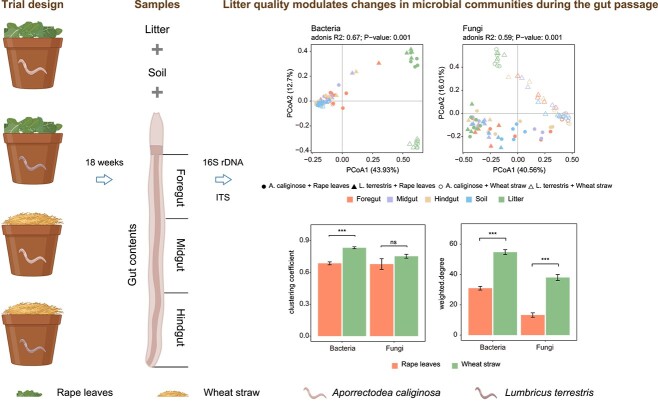

## Introduction

Earthworms are among the most important decomposer animals in terrestrial ecosystems, regulating nutrient cycling and stimulating primary production due to processing soil and mixing litter materials into the soil [1, 2]. Earthworms can break down litter materials by themselves, but also rely on microorganisms in their gut that produce digestive enzymes for degrading structural compounds such as chitin, and converting the ingested food resources into bioavailable molecules [2, 3].

The composition of gut microorganisms in earthworms varies with the soil they ingest, but also differs among earthworm species due to specific conditions in their gut [4–6]. Compared to soil, the gut environment of earthworms is characterized by lower oxygen availability, favoring anaerobe bacteria [2]. Higher concentrations of nitrogen, water-soluble carbon compounds and nitrite favor the proliferation and activity of denitrifying and methanogenic bacteria [7–9]. The gradual decrease in the concentration of nitrogen, water-soluble carbon compounds and nitrite from foregut to hindgut likely also changes gut bacterial and fungal communities during the gut passage [8, 10, 11]. These changes may be more pronounced in fungi than in bacteria as fungi are less adapted to the anaerobic gut environment than bacteria [2, 12].

Earthworm species are categorized into different ecological groups, which live and feed in different soil layers. Endogeic earthworm species live and feed in the mineral soil, while anecic earthworm species feed on surface litter which they pull into their vertical burrows in the soil and mix it with mineral soil [13, 14]. These differences between earthworm ecological groups likely result in unique gut microbiomes. Moreover, the residence time of ingested material in the gut and the efficiency of carbon assimilation differ between earthworm species. For instance, the food transit time in the gut of the anecic species *Lumbricus terrestris* has been estimated to be about 8 hours, while that of the endogeic species *Aporrectodea caliginosa* is in the range of 1–3 hours [15, 16]. The longer gut transit in *L. terrestris* is associated with higher food assimilation efficiency, which may reach 43–55%, while in endogeic species it is much lower, typically lower than 3% [17–20]. Therefore, the influence of earthworm ecological groups on gut microbiota composition may surpass that of the food ingested [21].

Litter quality is a pivotal factor shaping bacterial and fungal communities [22, 23]. Litter of low C-to-N ratio is more susceptible to degradation and exhibits a higher bacteria-to-fungi ratio [24]. Conversely, litter of high C-to-N ratio and lignin concentration favors the colonization by fungi, Gram-negative bacteria and oligotrophic microbial groups [24–26]. Low-quality and low nitrogen concentration of litter may result in strong nutrient competition and microbial niche differentiation, ultimately leading to more complex microbial communities [27]. As complex microbial communities are more stable and less responsive to environmental change [28], microbial communities of low-quality litter resources may change less during the gut passage of earthworms than those of high-quality litter. However, studies on the effect of litter quality on changes in microbial community composition during the gut passage of earthworms of different ecological groups are lacking.

We conducted a full factorial microcosm experiment to investigate changes in bacterial and fungal community composition during the gut transit in earthworms of different ecological groups (anecic and endogeic) and litter quality. We hypothesized that during the gut passage of earthworms, (1) changes in alpha diversity and community composition are more pronounced in fungi than in bacteria; (2) low-quality litter supports higher diversity and more complex microbial communities than high-quality litter, resulting in more complex microbial networks that change less during the earthworm gut passage; and (3) changes in microbial community composition during the gut passage are more pronounced in the anecic *L. terrestris* than in the endogeic *A. caliginosa* due to the different resources they use and their different gut transit times.

## Materials and methods

### Microcosm experiment

We set up a microcosm experiment investigating two factors each with two levels, litter type (rape leaves and wheat straw) and earthworm species (*A. caliginosa* and *L. terrestris*). Based on C-to-N ratio (rape leaves 12.3 ± 0.7, wheat straw 134.4 ± 0.8) and lignin content (rape leaves 5.1 ± 0.7%, wheat straw 20.8 ± 1.8%), rape leaves were ranked as high and wheat straw as low food quality. Earthworms were collected from a meadow close to the University of Göttingen, Germany (51°32′17.52″N, 9°56′12.12″E). The two earthworm species are common in central European ecosystems including forests, grasslands and arable systems. More detailed information on earthworm feeding preferences is given in Section S1. Surface soil (0-20 cm) was taken from an agricultural field located in Relliehausen, Lower Saxony, Germany (51°46′42.4"N, 9°41′42.7"E), which is characterized as Luvisol on loess with loamy texture. The soil was sieved through 4 mm mesh to remove larger plant residues and stones.

The microcosms consisted of PVC tubes (10 cm diameter, 17 cm height), with the bottom covered with a 200 μm mesh and the top surrounded by a transparent plastic sheet extending 10 cm above the microcosm tube to prevent earthworms from escaping. Each microcosm was filled with a mixture of 633 g fresh weight sieved soil and 324 g expanded clay (Bellandris Blähton, Sagaflor AG, Kassel, Germany). The expanded clay was added to improve soil structure. Soil moisture was kept at 70% of the maximum water holding capacity throughout the experiment by adding distilled water. Three grams of dry litter was added to the soil surface and an additional 1 g of the respective litter was added every 3 weeks thereafter to ensure continuous litter resource supply. A total of 20 microcosms were set up with five replicates each (2 litter treatments × 2 earthworm species × 5 replicates). Only juvenile earthworms were used to allow growth during the experiment. Five individuals of *A. caliginosa* and *L. terrestris* were inoculated into each microcosm of the respective treatments; the initial average total fresh earthworm biomass per microcosm was 1.63 ± 0.01 and 2.21 ± 0.01 g for *A. caliginosa* and *L. terrestris*, respectively. Adult and juvenile earthworms were identified to species using the determination key in Sims and Gerard [29]. The juveniles were classified based on pigmentation, setae arrangement and prostomium shape, and knowledge on the earthworm species present at our sampling site. The description of earthworm species identification is provided in Section S2. Microcosms were placed in darkness in a climate chamber at 20 ± 2°C and 70% humidity, watered four times a week based on gravimetric determination of the water loss and randomized twice per week. After 18 weeks, microcosms were destructively sampled.

### Sampling

Litter materials were collected from the soil surface, lyophilized at −70°C and stored at −20°C for further analysis. Earthworms were separated from soil by hand, washed and killed by placement in 70% ethanol for 2 min. Then, earthworms were placed on sterile iced tissue paper for 3 min to remove adhering ethanol, and then transferred to a sterile dissecting plate. Their gut was removed with a sterile scalpel and evenly divided into three parts (the foregut, midgut, and hindgut) [30, 31]. The gut content of four to five individuals from the same treatment were pooled to obtain enough material for DNA extraction. Thereafter, the gut contents were lyophilized at −70°C (Section S3) and stored at −20°C for further analysis. Bulk soil was sieved at 2 mm and stored at −20°C until further analysis.

### DNA extraction and PCR amplification

Total DNA was extracted from 250 mg dried foregut, midgut, hindgut, soil and litter using the DNeasy PowerSoil Pro Kit (Qiagen, Hilden, Germany). The primers and the process of PCR amplification are reported in the supplementary (Section S4). Amplification of PCR products were checked using QIAxcel (Qiagen), purified using QIAquick 96 PCR Purification Kit. Purified PCR products were quantified by Qubit 2 (Thermo Fisher Scientific, Waltham, MA, USA). ZymoBIOMICS microbial community standards (Zymo Research Corp, Irvine, CA, USA) and Mycobiome Genomic DNA Mix (ATCC, Manassas, VA, USA) were used to evaluate the efficiency of the PCR method and DNA extraction (Fig. S1).

### Illumina MiSeq sequencing

Purified PCR products were sent to Göttingen Genome Sequencing Laboratory (G2L, University of Göttingen) for library construction on the Illumina MiSeq platform (paired-end, 2 × 250 bp). Quality control of raw data is given in Section S5, and further information on sequencing data is provided in Table S1.

Operational taxonomic units (OTUs) were clustered with 97% similarity cutoff using UPARSE (version 10.0; http://drive5.com/uparse/). The phylogenetic affiliation of each 16S rRNA gene sequence and ITS were analyzed by the uclust algorithm (http://www.drive5.com/usearch/manual/uclust_algo.html) against the SILVA (SSU138.1; https://www.arb-silva.de) 16S rRNA database and UNITE ITS database using a confidence threshold of 80%. FUNGuild was used to ascribe sequences to functional groups of fungi [32].

### Statistical analysis

OTUs were rarefied based on the minimum number of sample sequences in R using the “vegan” package [33]. Abundance-based coverage estimator (ACE) and Shannon index were calculated for estimating alpha diversity using normalized OTUs. Linear mixed effects models were used to evaluate the effects of litter type (rape leaves, wheat straw), earthworm species (*A. caliginosa, L. terrestris*) and sample type (foregut, midgut, hindgut, soil, litter) on ACE and Shannon index, with sample ID included as a random factor, using the “lme4” package in R [34].

Principal coordinates analysis (PCoA) based on Bray–Curtis dissimilarity was conducted to examine the effect of earthworm species, litter type and sample type on microbial communities (beta diversity). We then run permutational multivariate analysis of variance using the “vegan” package to examine whether variation in microbial communities could be explained by earthworm species, litter type, and sample type.

The relative abundance of taxa was calculated at phylum level; phyla with a relative abundance <0.1% were classified as “Others”. OTUs that were significantly different between sample types were analyzed based on the ANCOM-II method. Two methods (ANCOM-II and ALDEx2) were used to analyze differences in the abundance of bacteria and fungi between earthworm species and litter types (detailed description in Section S6).

We constructed four microbial networks: (i) Bacterial and fungal OTUs from the combined data of the gut, soil and litter; (ii) significantly different bacterial and fungal OTUs from the combined data of the gut, soil and litter; (iii) bacterial and fungal OTUs from the combined data of the foregut, midgut and hindgut of earthworms; and (iv) bacterial and fungal OTUs from the foregut, midgut, hindgut, soil and litter. OTUs with a relative abundance >0.1% were used for Spearman correlation analysis using the R package “WGCNA” [35]. Only significantly correlated OTUs (*P* < .05 and |r| > 0.6) were used to construct co-occurrence networks. Node, edge and parameters such as clustering coefficient and weighted degree were extracted in Gephi (version 0.10) from the *gexf* file generated by the “igraph” package in R [36]. For visualization of pairwise differences, Tukey's honestly significant difference (HSD) test was used as implemented in the R package “emmeans” [37].

## Results

### Bacterial and fungal diversity

**Figure 1:**
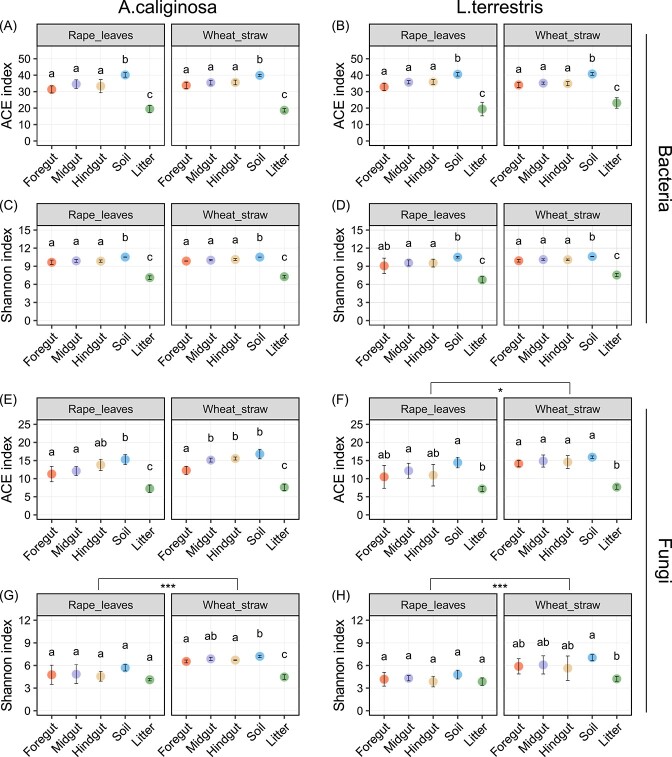
Bacterial (A, B, C, D) and fungal (E, F, G, H) alpha diversity in the foregut, midgut, and hindgut of two earthworm species [*Aporrectodea caliginosa* (left panels) and *Lumbricus terrestris* (right panels)], soil and two litter types used as food substrate (rape leaves or wheat straw) as indicated by the ACE and Shannon index (mean ± SE). Different letters indicate significant differences between means (Tukey's HSD test, *P* < .05). Asterisks indicate significant differences between litter types nested within earthworm species (^*^P < .05 and ^*^^*^^*^P < .001; Tukey's HSD test).

Bacterial alpha diversity (ACE and Shannon indices) mainly varied with sample type (Fig. 1A–D, Table S2). In both the anecic species *L. terrestris* and the endogeic species *A. caliginosa*, the ACE and Shannon indices did not differ significantly among gut sections, ranging from 31.4 to 35.7 and from 9.1 to 10.1, respectively. However, both the ACE and Shannon indices of earthworm gut bacteria were significantly lower than those of soil (39.9-40.7 and 10.5-10.6, respectively) but higher than those of litter (18.7-23.1 and 6.8-7.5, respectively).

Unlike bacteria, both ACE and Shannon indices of fungi differed significantly with litter type (Fig. 1E–H, Table S3). The ACE and Shannon indices in earthworm gut sections and in soil in the treatment with wheat straw were significantly higher than in the treatment with rape leaves, with this being particularly evident for the Shannon index. However, as indicated by the Shannon index, the effect of litter type depended on sample type (significant litter type × sample type interaction; Table S3). In the wheat straw treatment, the Shannon index in earthworm gut sections and in soil was significantly higher than in litter, while it did not differ between sample types in the rape leaves treatment (Fig. 1G, H). Additionally, the Shannon index also varied significantly with earthworm species, being generally higher in the *A. caliginosa* than the *L. terrestris* treatment (Fig. 1G, H, Table S3).

### Microbial community composition

**Figure 2:**
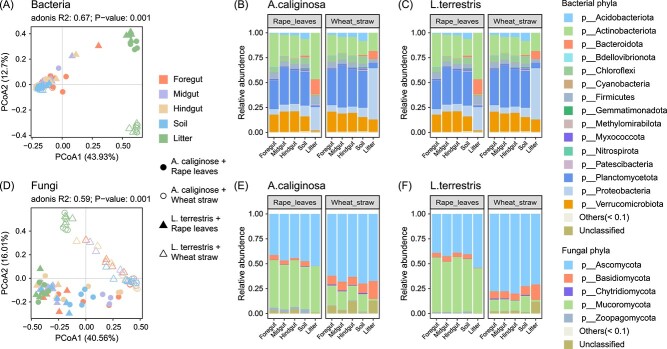
PCoA plot showing beta-diversity of bacteria (A) and fungi (D) in the foregut, midgut, hindgut, soil, and litter in different litter and earthworm species treatments, i.e. *Aporrectodea caliginosa* with rape leaves (solid circles), *A. caliginosa* with wheat straw (empty circles), *Lumbricus terrestris* with rape leaves (solid triangles) and *L. terrestris* with wheat straw (empty triangles) based on Bray-Curtis distances. Relative abundance of bacterial (B, C) and fungal phyla (E, F) in the foregut, midgut and hindgut of the two earthworm species, soil and two litter types used as food substrate (rape leaves or wheat straw).

Bacteria were successfully annotated to 15 phyla (Fig. 2A–C). Bacterial community composition varied significantly with litter type, earthworm species and sample type as well as each of the two factor interactions (Fig. 2A, Table S4). Generally, Planctomycetota, Actinobacteriota, Verrucomicrobiota, Proteobacteria and Firmicutes were the dominant bacterial phyla in both earthworm gut sections and soil. However, the relative abundances of dominant bacteria varied among gut sections. For example, Actinobacteriota had the highest relative abundance in the foregut (32.1%) and decreased towards the midgut (21.4%) and hindgut (19.8%). Conversely, oligotrophic bacteria, such as Planctomycetota and Verrucomicrobiota, increased from 24.0% to 36.0% and from 14.6% to 19.1% from foregut to hindgut, respectively. Litter was dominated by similar bacterial phyla as the gut sections and soil, but Planctomycetota were less abundant, whereas Bacteroidota were more abundant (rape leaves 16.5%; wheat straw 9.3%). Further, in wheat straw the relative abundance of Proteobacteria was particularly high (51.3%) and considerably exceeded that in rape leaves (25.4%), gut sections (average of 7.6%) and soil (10.3%).

Fungi were successfully annotated to five phyla, with more than 95% being saprotrophs (Fig. 2D–F, Fig. S2). Similar to bacteria, fungal community composition varied significantly with litter type, earthworm species, and sample type as well as the two factor interactions between litter type and earthworm species, and that between litter type and sample type (Table S5). Differences between litter type treatments were most pronounced between rape leaves and wheat straw (Fig. 2D). Further, the effect of litter type was more pronounced in *L. terrestris* than in *A. caliginosa* treatments mainly due to the higher relative abundance of Ascomycota in the wheat straw treatment with *L. terrestris*. Generally, the relative abundance of Ascomycota and Basidiomycota in wheat straw was 70.9% and 9.9%, respectively, which was higher than in rape leaves (44.8% and 3.5%, respectively). Conversely, the relative abundance of Mucoromycota was lower in wheat straw (12.5%) than in rape leaves (48.8%).

### Bacterial and fungal networks

**Figure 3:**
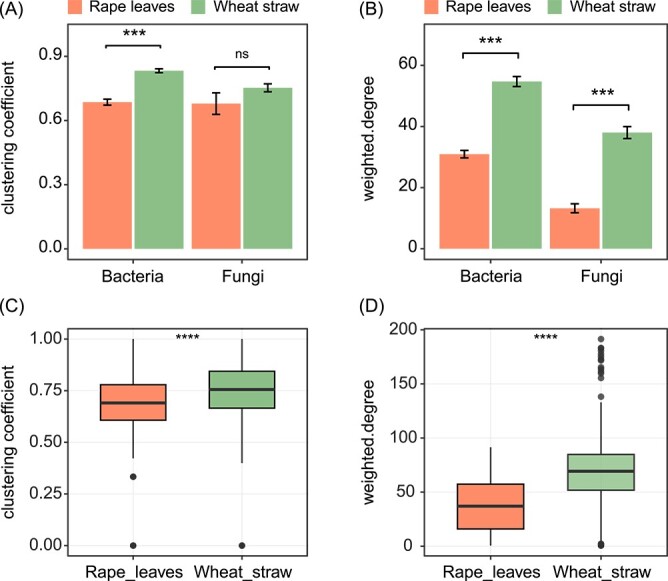
Bar plots on clustering coefficient (A) and weighted degree (B) of networks based on bacterial and fungal OTUs (pooled for the two earthworm species studied, i.e. *Aporrectodea caliginosa* and *Lumbricus terrestris*) in rape leaves and wheat straw treatments (means ± SE). Box plots on clustering coefficient (C) and weighted degree (D) of networks based on combined bacterial and fungal OTUs (pooled for the two earthworm species studied) in rape leaves and wheat straw treatments (means ± SE). Asterisks indicate significant differences, ^*^^*^^*^P < .001, ns *P* > .05 (Tukey's HSD test).

When analysing the full set of sample types (earthworm gut sections, soil and litter), the microbial associations (number of edges) in both bacterial and fungal networks were much higher in wheat straw than rape leave treatments (bacterial network 6432 vs 3645, fungal network 3670 vs 538, respectively; Fig. S3a,b). Similarly, competitive interactions (edges associated with negative correlations) were more frequent in wheat straw treatments (bacterial network 42.4% vs 34.5%, fungal network 27.8% vs 17.1%, respectively). For bacterial OTUs, fungal OTUs as well as significantly different OTUs of bacteria and fungi, the network clustering coefficient and weighted degree of wheat straw treatments were significantly higher than those of rape leave treatments (Figs. 3A, B, S4, Table S6). In the combined network of bacterial and fungal OTUs, there were 235 and 307 total network nodes in rape leaves and wheat straw treatments, respectively (Fig. S5a). Competitive interactions were more pronounced in wheat straw treatments for all OTUs, bacterial OTUs, fungal OTUs and those connecting significantly different bacterial and significantly different fungal OTUs, with percentages of 44.9%, 50.4%, 27.8% and 20.0%, respectively, compared to rape leaves treatments with respective percentages of 34.8%, 36.5%, 17.1% and 12.8% (Fig. S5b). Furthermore, the average clustering coefficient and weighted degree of OTUs of microbial networks in wheat straw treatments were significantly higher than those in rape leaves treatments (by 0.1 and 37.3, respectively; Fig. 3C, D, Table S6).

In the earthworm gut (combined data of foregut, midgut and hindgut), the clustering coefficient and weighted degree of microbial communities were higher in wheat straw than in rape leaves treatments but this was only significant for the weighted degree (Fig. S6a, b, Table S7). Further, during the gut passage, microbial networks generally changed less in wheat straw than in rape leaves treatments (Fig. S7a–d). Specifically, in rape leaves treatments, the network clustering coefficient of microbial communities in the midgut of *A. caliginosa* (0.74) was significantly higher than that in the hindgut (0.53), but did not differ significantly from that in the foregut (0.66). Conversely, the network clustering coefficient of microbial communities in the foregut of *L. terrestris* in the rape leaves treatment (0.84) was significantly higher than in both the midgut (0.48) and hindgut (0.50). However, in wheat straw treatments, the network clustering coefficient and weighted degree did not differ significantly among gut sections in both *A. caliginosa* and *L. terrestris*. Additionally, in the rape leaves treatments, the clustering coefficient and weighted degree of microbial communities in the foregut of both earthworm species were closer to litter, whereas those in the hindgut were similar to soil. Conversely, in wheat straw treatments, the clustering coefficient and weighted degree of microbial communities in the gut of *L. terrestris* were similar to those in soil and significantly lower than those in litter.

## Discussion

As ecosystem engineer, earthworms regulate nutrient cycling in terrestrial ecosystems, mainly due to their ability to fragment and process large amounts of litter materials [1, 2, 6]. The gut microbiome acts as a “hidden” player in earthworm-mediated decomposition processes, potentially modifying the effects of earthworms on soil processes [6]. Understanding how litter quality affects microbial communities during the gut passage of earthworms from different ecological groups is essential for a deeper comprehension of the complex feeding interactions in soil systems. We found that microbial communities changed little during the gut transit of earthworms. Bacteria communities more closely resembled those in soil than those in litter, irrespective of the ecological group of earthworms. When earthworms were fed low-quality litter, bacterial alpha diversity was higher and microbial networks were more complex compared to when earthworms were fed high-quality litter. Notably, bacterial communities generally changed little during the gut passage, but this was somewhat less pronounced in the endogeic species *A. caliginosa* than in the anecic species *L. terrestris*. Overall, earthworm gut microbial communities were largely shaped by the surrounding soil rather than by the litter they were fed with. However, the quality of litter played a significant role in modulating these communities during the gut transit, but this depended on the ecological group of earthworms.

### Changes in microbial communities during the gut transit

Consistent with our first hypothesis, differences in microbial community composition among gut sections were more pronounced in fungi than in bacteria. This was particularly true for earthworms fed with low-quality litter, compared to those fed with high-quality litter. However, the changes in fungal community composition during the earthworm gut passage may not be due to digestion. A parallel study from the same experiment documented predominant contribution of bacteria-derived essential amino acids to earthworm nutrition (~60%), while fungal-derived resources contributed little (~10%) [38], suggesting that fungi are of minor importance for the diet of earthworms. Contrasting these findings, Wolter and Scheu [12] found fungi to be more sensitive to digestion processes during the gut passage of earthworms than bacteria and this is consistent with the fact that many bacteria are able to live as facultative anaerobes, whereas the majority of fungi needs an aerobic environment [23]. Consequently, low oxygen supply in the earthworm gut may exert a more pronounced effect on the fungal than the bacterial community [39]. However, regardless of earthworm species microbial communities in the earthworm gut more closely resembled those in soil than those in litter, presumably reflecting the ingestion of bulk soil hosting more diverse microbial communities dominated by bacteria compared to litter. Additionally, the reduced oxygen supply in the earthworm gut more closely resembles the microenvironment in soil aggregates rather than in litter, and this may also contribute to the gut microbial communities being more similar to those in soil than to those in litter. In fact, processes associated with low oxygen supply, such as dinitrogen emission, have been shown to be stimulated during the gut passage of soil through earthworms [7, 10]. Considering the partly anaerobic conditions, future studies may also sequence archaea to provide a more comprehensive understanding of changes in microbial communities during the gut passage of earthworms.

Microbial communities not only differed between the gut, soil and litter, but changed during the gut passage. For example, from foregut to hindgut, the relative abundance of oligotrophic bacteria (e.g., Planctomycetota and Verrucomicrobiota) increased, while that of copiotrophic bacteria (Actinobacteriota) decreased. In the earthworm foregut, large amounts of mucus is added to the food substrate ingested, whereas in the hindgut carbon compounds digested during the gut passage are resorbed [12, 40, 41]. These processes likely favor copiotrophic bacteria flourishing in resource-rich environments in the foregut, whereas in the hindgut oligotrophic bacteria adapted to resource-poor environments are favored [10, 42, 43].

However, overall, microbial communities changed little during the gut passage. This suggests that the differences between microorganisms in the gut and those in soil and litter are mainly due to earthworms selectively feeding on different food materials. In fact, earthworms are known to be able to sense and selectively feed on litter materials of different quality as well as different fungal species [44–46]. Although microorganisms are of high nutritional value due to their protein content by far exceeding that of litter and soil, microorganisms may pass through the gut of earthworms without digestion as their digestion may require specific adaptations, such as grinding mechanisms or specific enzymes, which appear to be absent or inefficient in the gut of earthworms [10, 46]. Further, the gut passage through earthworms may be too fast (2–24 h) to allow microorganisms to reproduce and build up populations [17, 47, 48].

The small changes in microbial diversity and composition during the earthworm gut transit indicates that live microorganisms contribute little to the diet of earthworms. This is supported by previous studies showing that microbial biomass and bacterial cell numbers change little during the earthworm gut passage [48, 49]. Combined with the high reliance of microbial essential amino acids in the studied earthworms [38], this suggests that rather than digesting live microorganisms, earthworms rely on microbial residues or proteins released by microorganisms during the gut passage [12, 48]. However, as this study is based on DNA analyses, it does not provide insight into the activity and growth of gut microorganisms. Future studies employing RNA sequencing are needed to provide a more comprehensive understanding of changes in the activity of microorganisms during the gut transit through earthworms. Nevertheless, our results suggest that rather than changing microbial communities during the gut passage, earthworms are likely to affect microbial communities in soil predominantly by mixing microorganisms of different microhabitats, such as litter and soil, spreading these microorganisms across soil layers via bioturbation and by providing unique growth conditions for microorganisms in earthworm casts [2, 50].

### Effect of litter quality on changes in microbial networks during the gut transit

Consistent with our second hypothesis, low-quality wheat straw exhibited higher alpha diversity, complexity and stability of microbial networks compared to high-quality rape leaves. Likewise, Sun et al. found higher microbial diversity in the earthworm gut when fed on low-quality wheat straw (C-to-N ratio of 39) than on high-quality peanut litter (C-to-N ratio of 16) [51]. Yang et al. also found more complex bacterial networks in in the casts of earthworms when fed on winter wheat (C-to-N ratio of 101) than on sainfoin (legume) litter (C-to-N ratio of 22) [52]. High-quality resources favor fast growing microbes, leading to their dominance [22, 53] and to reduced microbial diversity and network stability. Conversely, low-quality litter harboring diverse and complex microbial communities is likely to be associated with high competition for resources, particularly nitrogen, resulting in more pronounced niche differentiation and increased complexity of microbial networks [54]. In microbial networks, competitive (negative correlations) rather than cooperative (positive correlations) interactions predominate and this likely contributed to the highly diverse microbial communities in our study [54]. Compared to rape leaves, the increased number of negatively correlated edges in wheat straw treatments suggests that low-quality litter promotes competitive interactions, thereby enhancing community interactions and contributing to network stability. Moreover, network stability in the earthworm gut can be assessed through parameters like weighted degree and clustering coefficient [52, 55–57]. Weighted degree refers to the combination of the degree (number of edges) and strength (sum of the edge weights) for a given node. Clustering coefficient represents the degree to which the nodes tend to cluster together. Therefore, complex networks with high connectivity (high weighted degree and clustering coefficient) typically are more robust to environmental changes than less complex networks [28, 58, 59] and this may also have contributed to the fact that microbial communities changed less during gut transit in the wheat straw than the rape leaves treatment.

Due to high resource quality associated with high nitrogen concentration, rape leaves are preferentially fed by earthworms compared to wheat straw [38]. However, due to denitrification processes during the gut passage through earthworms, the availability of nitrogen is decreasing from foregut to hindgut [10]. This shift likely alters the competitive dynamics within the gut microbial community, likely disadvantaging microorganisms adapted to high nitrogen environments. This is supported by the fact that in the rape leaves treatment, the microbial network in the foregut closely resembled that of the nitrogen-rich rape leaves, while in the hindgut it more closely resembled that of the relatively nutrient poor soil. Additionally, the microbial network in the gut of both earthworm species in wheat straw treatments was more similar to that in soil than in litter (except for the clustering coefficient in *A. caliginosa*), and this was consistent with our results on alpha diversity.

### Effect of earthworm species on gut microbes

The two earthworm species used in our study represent two different ecological groups, with *A. caliginosa* representing endogeic and *L. terrestris* anecic species [13]. Endogeic species predominantly feed on mineral soil, whereas anecic species predominantly feed on litter [2]. Contrary to our third hypothesis, bacterial and fungal communities in the gut of the two earthworm species generally differed little, suggesting that differences in their feeding behavior may be smaller than previously assumed. Supporting this assumption, recent studies documented similar burrowing activities of *A. caliginosa* and *L. terrestris* suggesting that the former may better be classified as epi-anecic species [14, 60]. In fact, it has been documented that *A. caliginosa* benefits markedly from high-quality litter material and selectively feeds on it [61–63]. However, the limited space and food substrate in our microcosms may also have contributed to the similar gut microorganisms of the two species.

Despite generally small differences in gut microbial communities in the two earthworm species, minor differences occurred, e.g. fungal alpha diversity in the gut was higher in the anecic *L. terrestris* than in the endogeic *A. caliginosa* and this was more pronounced in wheat straw than rape leaves treatments. This suggests that *L. terrestris* depends less on high-quality litter and ingests low-quality litter such as wheat straw which is consistent with earlier studies [64, 65]. Similarly, fungal beta diversity was higher in the gut of *L. terrestris* than in *A. caliginosa* and was more similar to that in litter. This agrees with behavioral differences of the two earthworm species as *L. terrestris* mixes microorganisms in litter and soil more vigorously due to its vertical burrowing activity than *A. caliginosa*, which contributes to the homogenization of microbial communities and their functioning across soil depths.

## Conclusion

Building on the diet-host-gut microorganism interaction framework [66], our study provided a comprehensive understanding of the role of litter resource quality on gut microorganisms of earthworms and their changes during the gut transit. Overall, microbial communities in the gut of earthworms are shaped by both litter resource quality and earthworm ecological group. However, generally, microbial communities in the earthworm gut were predominantly shaped by the soil rather than the litter ingested and changed little during the gut transit. This likely reflects the predominant ingestion of soil rather than litter by both earthworm species studied, with most microorganisms resisting digestion processes during the gut passage. Additionally, the ingestion of low-quality litter modulated microbial communities in the gut of earthworms by increasing the diversity and complexity of microbial networks. Together, our results highlight that gut microbial communities of earthworms are predominantly shaped by the substrate ingested, with soil microorganisms playing a more important role than litter microorganisms. Further, litter quality functions as key factor influencing microbial communities in the gut of earthworms. Although earthworms ingest and process large amounts of microorganisms, digestion processes overall only little affect the ingested microbial communities suggesting that living microorganisms form only a small fraction of the diet of earthworms.

## Supplementary Material

Fig_S1_ycae171

Fig_S2_ycae171

Fig_S3_ycae171

Fig_S4_ycae171

Fig_S5_ycae171

Fig_S6_ycae171

Fig_S7_ycae171

Supplementary_20241217_ycae171

## Data Availability

All raw sequencing data have been deposited in the NCBI Sequence Read Archive (PRJNA1117918 and PRJNA1117914).
