# Supplementary material for: Litter quality modulates changes in bacterial and fungal communities during the gut transit of earthworm species of different ecological groups
Source: ISME Commun. 2024 Dec 26;5(1):ycae171. doi: 10.1093/ismeco/ycae171 (PMC11778916; doi:10.1093/ismeco/ycae171)
Supplement: Fig_S1_ycae171 [file fig_s1_ycae171.docx]

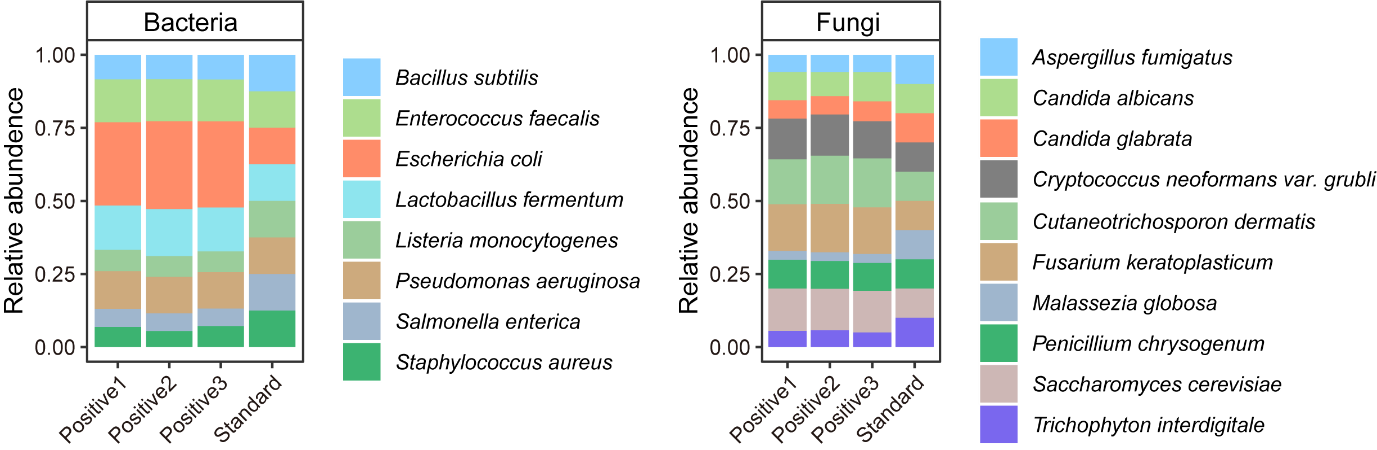


**Fig. S1** Relative abundance of bacterial/fungal species in the positive samples and bacterial (ZymoBIOMICS™ Microbial Community Standard)/ fungal (ZymoBIOMICS™ Microbial Community Standard) standards. The bacterial/fungal communities of positive samples showed concordance between with the suppliers’ specifications. *Escherichia coli* mistakenly classified as *Shigella*. *Shigella* and *Escherichia coli* are very similar that *Shigella* could be classified as a subgenus or a pathovar within *E. coli* rather than a distinct genus.
