# Supplementary material for: Litter quality modulates changes in bacterial and fungal communities during the gut transit of earthworm species of different ecological groups
Source: ISME Commun. 2024 Dec 26;5(1):ycae171. doi: 10.1093/ismeco/ycae171 (PMC11778916; doi:10.1093/ismeco/ycae171)
Supplement: Fig_S2_ycae171 [file fig_s2_ycae171.docx]

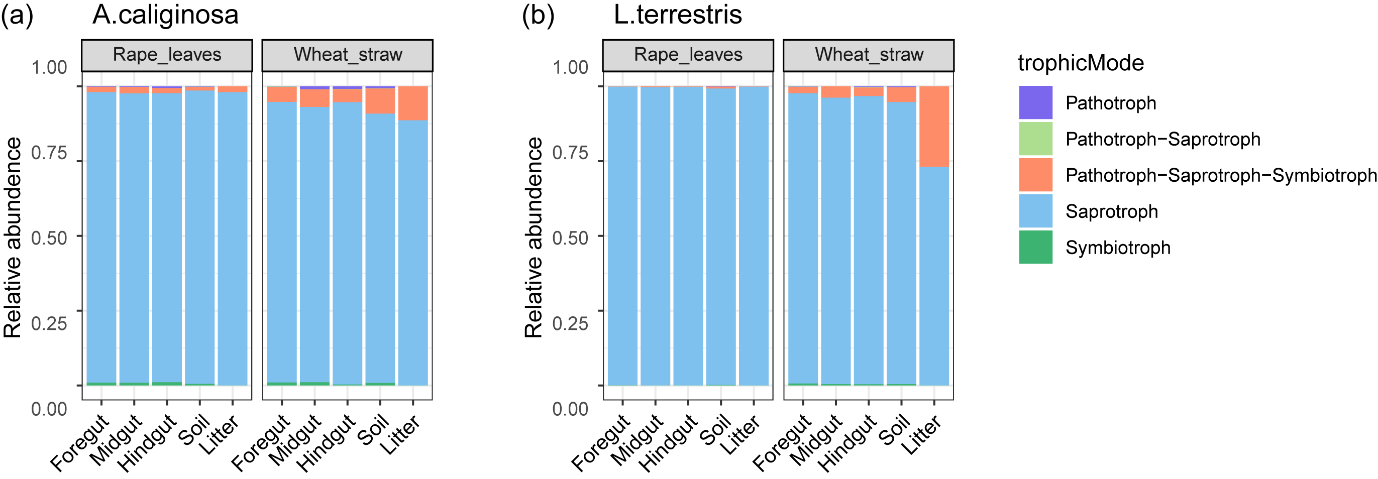


**Fig. S2** Relative abundance (means) of fungal trophic mode in the foregut, midgut and hindgut of *Aporrectodea caliginosa* (a) and *Lumbricus terrestris* (b), as well as in soil and two litter types used as food substrate (rape leaves or wheat straw).
