# Supplementary material for: Litter quality modulates changes in bacterial and fungal communities during the gut transit of earthworm species of different ecological groups
Source: ISME Commun. 2024 Dec 26;5(1):ycae171. doi: 10.1093/ismeco/ycae171 (PMC11778916; doi:10.1093/ismeco/ycae171)
Supplement: Fig_S3_ycae171 [file fig_s3_ycae171.docx]

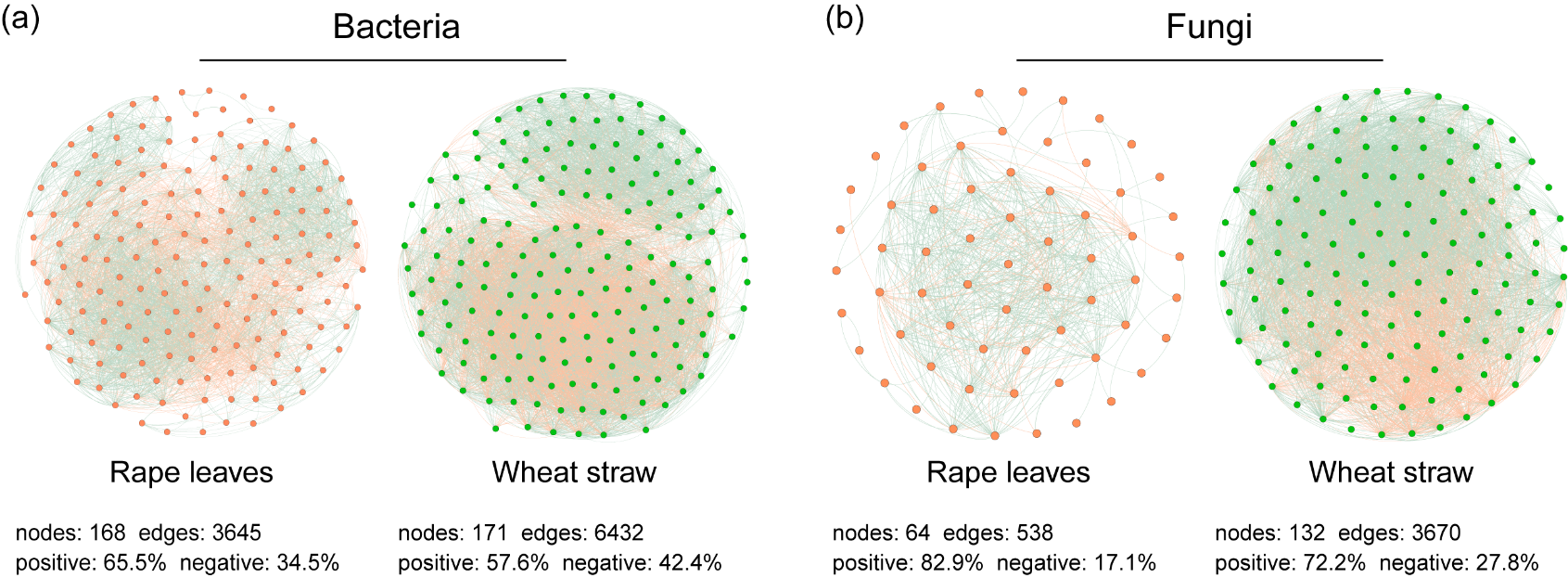


**Fig. S3** Co-occurrence networks based on bacterial (a) and fungal (b) OTUs in the gut of earthworms (pooled for the two species studied, i.e. *Aporrectodea caliginosa* and *Lumbricus terrestris*) fed with different litter materials (rape leaves and wheat straw); orange links, negative relationship; green links, positive relationship; orange nodes, OTUs from rape leaves treatments; green nodes, OTUs from wheat straw treatments.
