# Supplementary material for: Litter quality modulates changes in bacterial and fungal communities during the gut transit of earthworm species of different ecological groups
Source: ISME Commun. 2024 Dec 26;5(1):ycae171. doi: 10.1093/ismeco/ycae171 (PMC11778916; doi:10.1093/ismeco/ycae171)
Supplement: Fig_S4_ycae171 [file fig_s4_ycae171.docx]

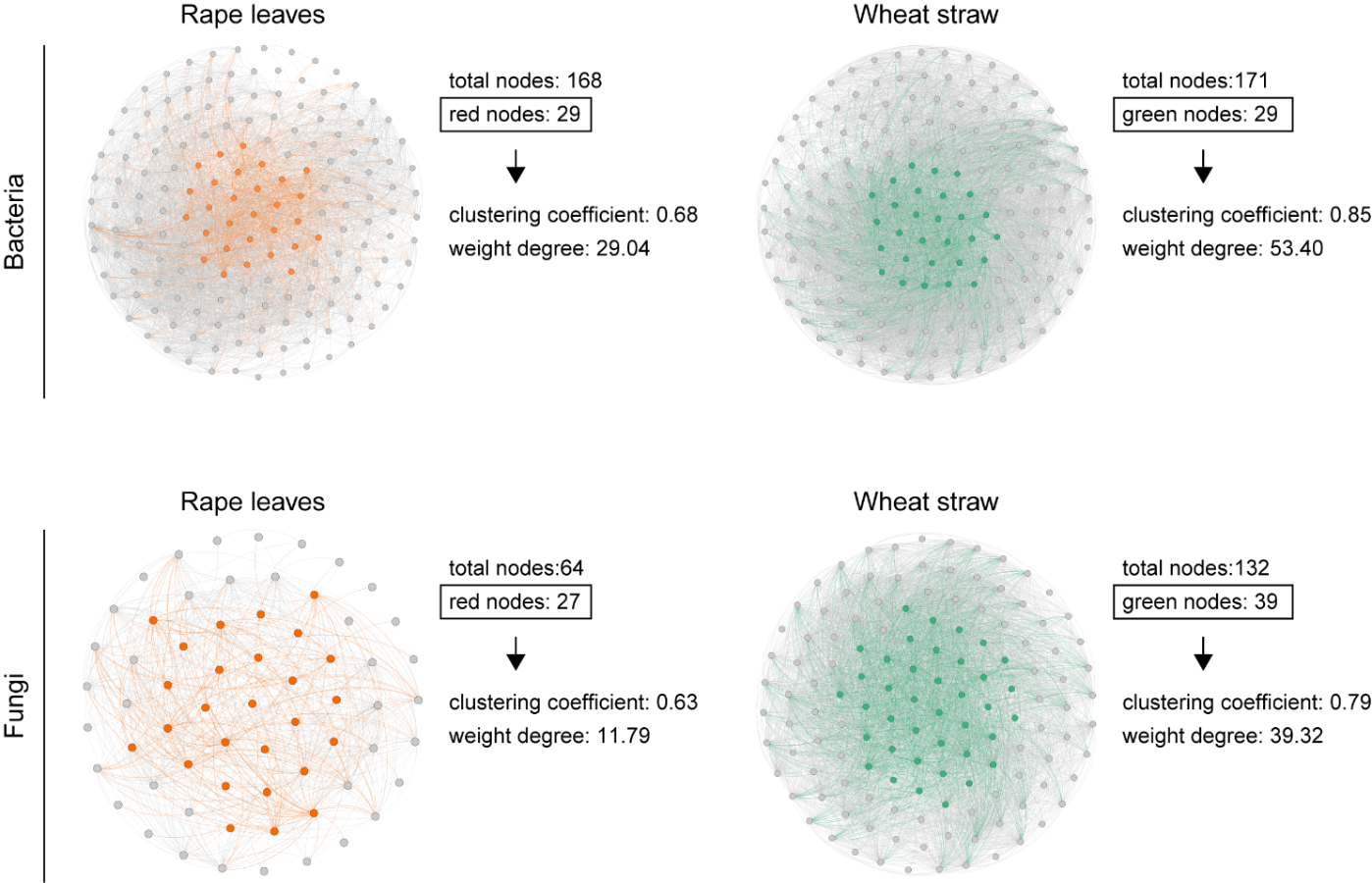


**Fig. S4** Co-occurrence network of significantly different OTUs of bacteria and fungi between rape leaves and wheat straw treatments; orange nodes represent significantly different OTUs from rape leaves treatments, green nodes represent significantly different OTUs from wheat straw treatments, grey nodes represent not significantly different OTUs; the color of each line in the co-occurrence network corresponds to the color of the source node.
