# Supplementary material for: Litter quality modulates changes in bacterial and fungal communities during the gut transit of earthworm species of different ecological groups
Source: ISME Commun. 2024 Dec 26;5(1):ycae171. doi: 10.1093/ismeco/ycae171 (PMC11778916; doi:10.1093/ismeco/ycae171)
Supplement: Fig_S5_ycae171 [file fig_s5_ycae171.docx]

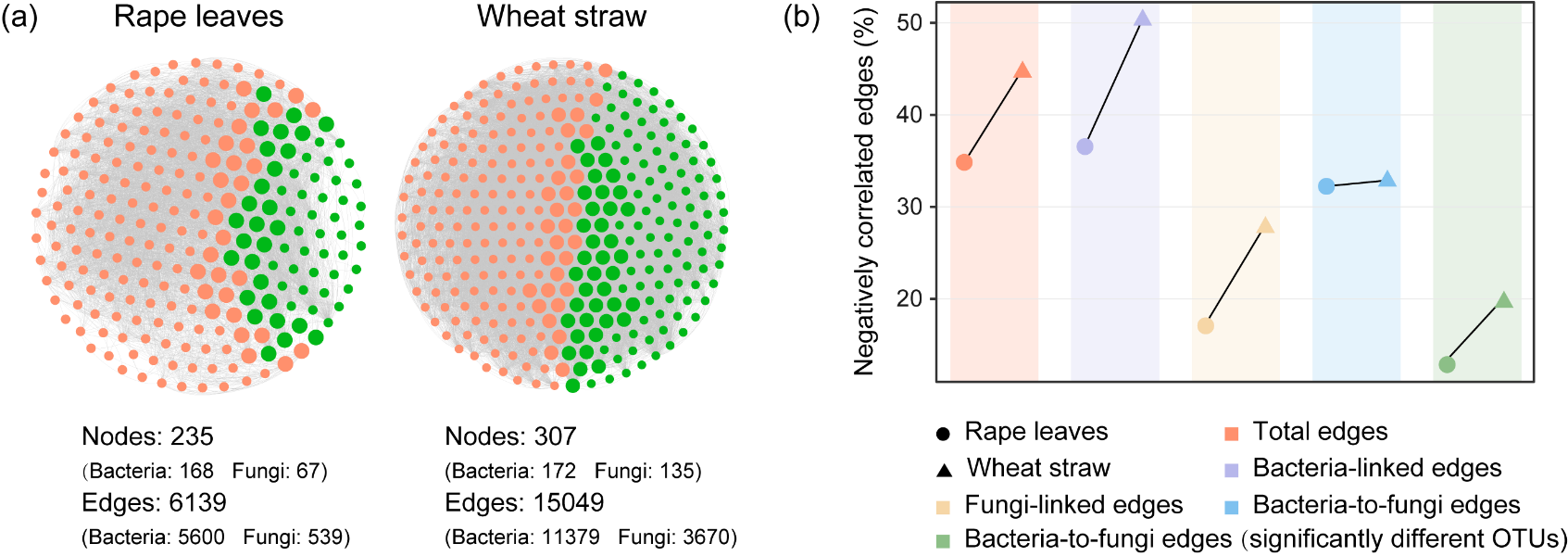


**Fig. S5** (a) Co-occurrence network of combined bacterial and fungal OTUs in the gut of earthworms (pooled for the two species studied, i.e. *Aporrectodea caliginosa* and *Lumbricus terrestris*) fed with rape leaves or wheat straw treatments; orange nodes, bacterial OTUs; green nodes, fungal OTUs; big and small nodes represent significantly different OTUs and not significantly different OTUs, respectively. (b) Percentage of negatively correlated edges in the networks; circles, rape leaves network; triangles, wheat straw network; orange area, all OTUs; purple area, bacterial OTUs; yellow area, fungal OTUs; blue area, connected bacterial and fungal OTUs; green area, significantly different bacterial OTUs and significantly different fungal OTUs.
