# Supplementary material for: Litter quality modulates changes in bacterial and fungal communities during the gut transit of earthworm species of different ecological groups
Source: ISME Commun. 2024 Dec 26;5(1):ycae171. doi: 10.1093/ismeco/ycae171 (PMC11778916; doi:10.1093/ismeco/ycae171)
Supplement: Fig_S6_ycae171 [file fig_s6_ycae171.docx]

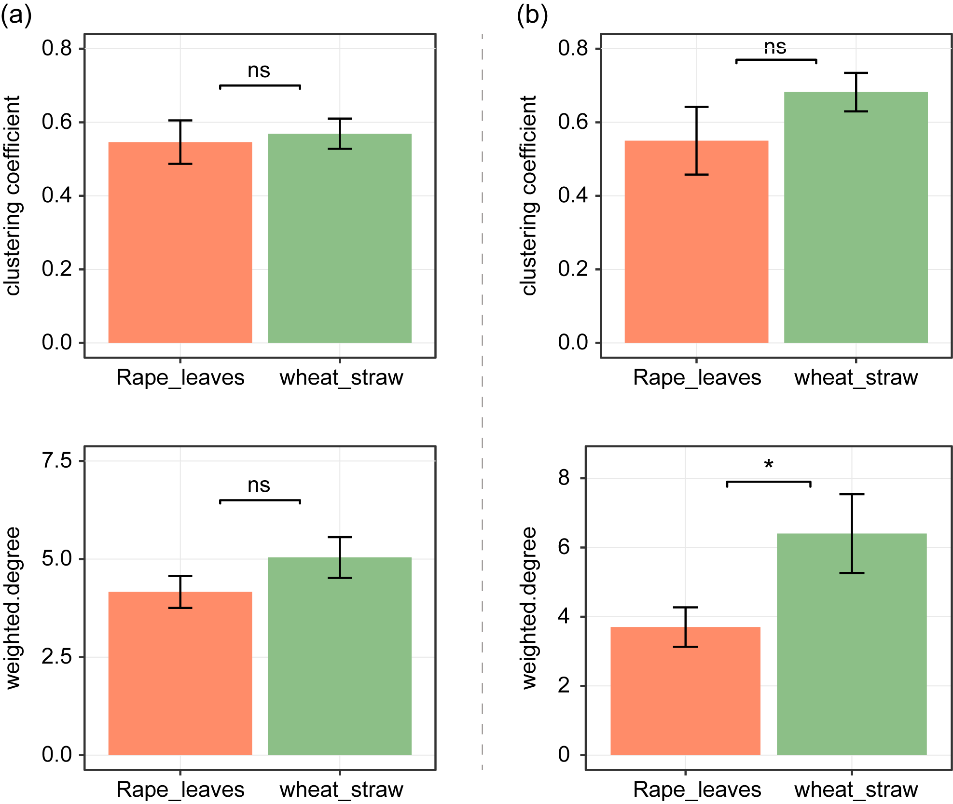


**Fig.S6** Bar plot on the clustering coefficient and weighted degree extracted from networks based on total OTUs (a) and significantly different OTUs (b) in the earthworm gut in rape and wheat straw treatments (means ± SE). Asterisks indicate significant differences, *p < 0.05 (Tukey's HSD test); n.s., not significant.
