# Supplementary material for: Litter quality modulates changes in bacterial and fungal communities during the gut transit of earthworm species of different ecological groups
Source: ISME Commun. 2024 Dec 26;5(1):ycae171. doi: 10.1093/ismeco/ycae171 (PMC11778916; doi:10.1093/ismeco/ycae171)
Supplement: Fig_S7_ycae171 [file fig_s7_ycae171.docx]

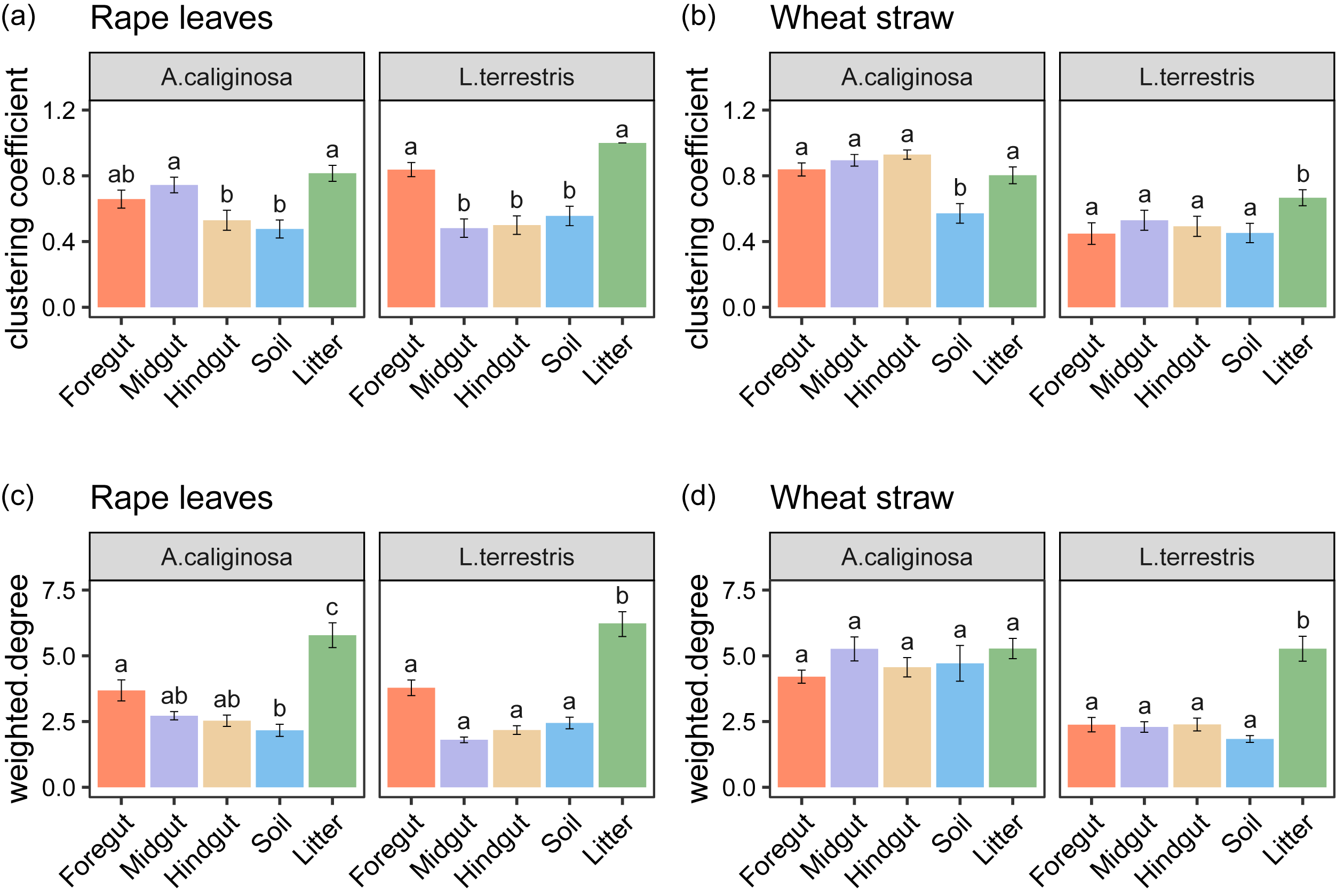


**Fig.S7** Bar plot on the clustering coefficient and weighted degree extracted from networks based on total OTUs in foregut, midgut and hindgut of two earthworm species (*Aporrectodea caliginosa* and *Lumbricus terrestris*), soil and two litter types used as food substrate [rape leaves (a, c) or wheat straw (b, d)] (means ± SE). Different letters indicate significant differences between means (Tukey's HSD test, p < 0.05).
