# Supplementary material for: Litter quality modulates changes in bacterial and fungal communities during the gut transit of earthworm species of different ecological groups
Source: ISME Commun. 2024 Dec 26;5(1):ycae171. doi: 10.1093/ismeco/ycae171 (PMC11778916; doi:10.1093/ismeco/ycae171)
Supplement: Supplementary_20241217_ycae171 [file supplementary_20241217_ycae171.docx]

**Section S1. Information on earthworm feeding preferences**

In a parallel study based on the same experiment, litter consumption of *L. terrestris* was significantly higher than that of *A. caliginosa* [1]. Furthermore, using compound-specific stable isotope analysis, we found that *L. terrestris* derived most of its essential amino acids from plant resources (~60%), whereas *A. caliginosa* derived most of its essential amino acids from bacterial resources (~55%). The litter-feeding behavior of *L. terrestris* confirms our classification as anecic earthworm species, while the high dependence of *A. caliginosa* on bacterial resources likely reflects its greater ingestion of soil, supporting its classification as endogeic species.

**Section S2. Earthworm species identification**

Live adult earthworms collected alongside juveniles at our sampling site were assigned to species using the key of Sims and Gerard [2], and live juveniles were assigned to species based on species characteristics such as pigmentation, setae arrangement and prostomium shape. *Lumbricus terrestris*, *Aporrectodea caliginosa*, *Allolobophora chlorotica*, *Aporrectodea rosea* and *Octolasion cyaneum* were the only species present. Ascription of juveniles to species and ecological groups was consistent with their feeding behavior and incorporation of amino acids of plant and bacterial origin as documented in Zhong et al. [1].

**Section S3. Intestinal content lyophilisation**

The gut contents were lyophilized at -70°C in a freeze-dryer. Specifically, the gut contents were placed in 2 mL centrifuge tubes with small holes in the tube caps to equalize pressure. The tubes were then lyophilized for 12 hours to ensure complete drying.

**Section S4. PCR amplification**

The V3 - V4 region of the bacteria 16S rDNA sequences were amplified by PCR using the primers Bact-0341 (5’- CCT ACG GGN GGC WGC AG -3’) and Bact-0785 (5’ - GGA CTA CHV GGG TAT CTA ATC C -3’) [3]. The ITS3 - ITS4 region of fungal internal transcribed spacer (ITS) sequences were amplified by PCR using the primers ITS3_KYO2 (5’- GAT GAA GAA CGY AGY RAA -3’) and ITS4 (5’ - GTC CTC CGC TTA TTG ATA TGC -3’) [4]. The PCR amplification conditions were as follows: Initial activation step at 95℃ for 3 min, 25 amplification cycles (denaturation at 98°C for 20 s, annealing at 55℃ (bacteria) or 48℃ (fungi) for 15 s, and extension at 72℃ for 1 min) and final extension step at 72℃ for 1 min. All PCR reactions for sequencing contained 5 μl template DNA, 0.75 μl of each primer (10 mM), 12.5 μl KAPA HiFi HotStart ReadyMix and 6 µl ultrapure H_2_O to make up a total volume of 25 μl per reaction.

**Section S5. Quality control of Illumina MiSeq sequencing**

Quality control of raw data was conducted using the following criteria: (i) trimming of low-quality reads (i.e., length < 50 bp, average quality score < 20), (ii) removal of nucleotide mismatches ≥ 2 in primer matching, and (iii) discarding sequences overlapping < 10 bp [5].

**Section S6. ANCOM-II and ALDEx2 for analyzing differences in the abundance of bacteria and fungi between earthworm species and litter types**

Specifically, we performed differential abundance analysis on the microbial community (OTU) using the ‘ANCOMBC’ package [6]. We excluded structural zeros, i.e., the taxa absent in one treatment but present in another. A pseudo-count of 1 was applied across the dataset to enable log transformation. Using the main function ANCOM, all additive log-ratios for each taxon were then tested for significance using Wilcoxon rank-sum tests, and p-values were FDR-corrected using the Benjamini-Hochberg (BH) method. Additionally, we passed the OTUs table to the *aldex* function of the ‘ALDEx2’ R package to perform Monte Carlo sampling of Dirichlet distributions with a uniform prior [7]. Each realization performed a CLR transformation and followed by Welch's t-tests on the transformed data. The resulting p-value was corrected using the BH procedure [8].

**Table S1**

Number and percentage of sequences for different length ranges in base pairs (bp) for both bacteria and fungi.

| Sequence length (bp) | Bacteria | | Fungi | |
| --- | --- | --- | --- | --- |
|  | Sequences number | Sequences percent | Sequences number | Sequences percent |
| 1-100 | 5630 | 0.13% | 3625 | 0.06% |
| 101-200 | 19124 | 0.44% | 4317 | 0.08% |
| 201-300 | 14603 | 0.33% | 15108 | 0.27% |
| 301-400 | 11888 | 0.27% | 4661621 | 83.58% |
| 401-500 | 4318197 | 98.79% | 829388 | 14.87% |
| 501-600 | 1716 | 0.04% | 63272 | 1.13% |

**Table S2**

F- and p-values of linear mixed effects models on the effect of litter type (rape leaves and wheat straw), earthworm species (*Aporrectodea caliginosa* and *Lumbricus terrestris*), sample type (foregut, midgut, hindgut, soil and litter) and their interaction on alpha diversity (ACE and Shannon index) of bacteria; numDf, numerator degrees of freedom; denDf, denominator degrees of freedom. Significant p-values are given in bold (p < 0.05).

| Response | Factor | numDf | denDf | F-value | p-value |
| --- | --- | --- | --- | --- | --- |
| ACE | Litter type (L) | 1 | 75 | 0.32 | 0.569 |
|  | Earthworm species (E) | 1 | 75 | 9.50 | **0.003** |
|  | Sample type (S) | 4 | 75 | 255.59 | **<0.001** |
|  | L x E | 1 | 75 | 0.04 | 0.831 |
|  | L x S | 4 | 75 | 0.75 | 0.559 |
|  | E x S | 4 | 75 | 0.64 | 0.633 |
|  | L x E x S | 4 | 75 | 2.42 | 0.055 |
| Shannon | Litter type (L) | 1 | 75 | 10.08 | **0.002** |
|  | Earthworm species (E) | 1 | 75 | 0.53 | 0.469 |
|  | Sample type (S) | 4 | 75 | 195.97 | **<0.001** |
|  | L x E | 1 | 75 | 7.09 | **0.009** |
|  | L x S | 4 | 75 | 0.96 | 0.435 |
|  | E x S | 4 | 75 | 0.46 | 0.767 |
|  | L x E x S | 4 | 75 | 0.35 | 0.845 |

**Table S3**

F- and p-values of linear mixed effects models on the effect of litter type (rape leaves and wheat straw), earthworm species (*Aporrectodea caliginosa* and *Lumbricus terrestris*), sample type (foregut, midgut, hindgut, soil and litter) and their interactions on alpha diversity (ACE and Shannon index) of fungi; numDf, numerator degrees of freedom; denDf, denominator degrees of freedom. Significant p-values are given in bold (p < 0.05).

| Response | Factor | numDf | denDf | F-value | p-value |
| --- | --- | --- | --- | --- | --- |
| ACE | Litter type (L) | 1 | 76 | 5914.83 | **<0.001** |
|  | Earthworm species (E) | 1 | 76 | 31.43 | **<0.001** |
|  | Sample type (S) | 4 | 76 | 1.07 | 0.303 |
|  | L x E | 1 | 76 | 2.10 | 0.151 |
|  | L x S | 4 | 76 | 1.82 | 0.134 |
|  | E x S | 4 | 76 | 1.69 | 0.161 |
|  | L x E x S | 4 | 76 | 0.81 | 0.524 |
| Shannon | Litter type (L) | 1 | 76 | 4444.77 | **<0.001** |
|  | Earthworm species (E) | 1 | 76 | 92.63 | **<0.001** |
|  | Sample type (S) | 4 | 76 | 12.70 | **<0.001** |
|  | L x E | 1 | 76 | 0.05 | 0.812 |
|  | L x S | 4 | 76 | 3.51 | **0.011** |
|  | E x S | 4 | 76 | 0.41 | 0.800 |
|  | L x E x S | 4 | 76 | 0.38 | 0.820 |

**Table S4**

F- and p-values of PERMANOVA on the effect of litter type (rape leaves and wheat straw), earthworm species (*Aporrectodea caliginosa* and *Lumbricus terrestris*), sample type (foregut, midgut, hindgut, soil, and litter) and their interactions on bacterial community composition. Df, degrees of freedom; SumOfSqs, sum of squares. Significant differences were given in bold (p < 0.05).

| Factor | Df | SumOfSqs | R^2^ | F-value | p-value |
| --- | --- | --- | --- | --- | --- |
| Litter type (L) | 1 | 0.96 | 0.04 | 12.97 | **0.001** |
| Earthworm species (E) | 1 | 0.49 | 0.02 | 6.64 | **0.001** |
| Sample type (S) | 4 | 9.92 | 0.48 | 33.37 | **0.001** |
| L x E | 1 | 0.29 | 0.01 | 3.93 | **0.005** |
| L x S | 4 | 2.04 | 0.10 | 6.88 | **0.001** |
| E x S | 4 | 0.64 | 0.03 | 2.18 | **0.012** |
| L x E x S | 4 | 0.38 | 0.01 | 1.29 | 0.178 |
| Residual | 76 | 5.64 | 0.27 |  |  |
| Total | 95 | 20.40 | 1 |  |  |

**Table S5**

F- and p-values of PERMANOVA on the effect of litter type (rape leaves and wheat straw), earthworm species (*Aporrectodea caliginosa* and *Lumbricus terrestris*), sample type (foregut, midgut, hindgut, soil, and litter), and their interactions on fungal community composition. Df, degrees of freedom; SumOfSqs, sum of squares. Significant differences were given in bold (p < 0.05).

| Factor | Df | SumOfSqs | R^2^ | F-value | p-value |
| --- | --- | --- | --- | --- | --- |
| Litter type (L) | 1 | 6.13 | 0.25 | 50.74 | **0.001** |
| Earthworm species (E) | 1 | 1.28 | 0.05 | 10.65 | **0.001** |
| Sample type (S) | 4 | 4.12 | 0.17 | 8.52 | **0.001** |
| L x E | 1 | 0.39 | 0.01 | 3.29 | **0.011** |
| L x S | 4 | 1.48 | 0.06 | 3.06 | **0.002** |
| E x S | 4 | 0.68 | 0.03 | 1.42 | 0.109 |
| L x E x S | 4 | 0.37 | 0.01 | 0.77 | 0.736 |
| Residual | 76 | 9.18 | 0.38 |  |  |
| Total | 95 | 23.67 | 1 |  |  |

**Table S6**

Pairwise comparisons of network attributes (clustering coefficient and weighted degree) calculated from bacterial OTUs, fungal OTUs and total OTUs in the foregut, midgut, hindgut, soil and litter in rape leaves and wheat straw treatments. SE, standard error; Df, degrees of freedom. Significant differences are given in bold (p < 0.05).

| OTUs | Network attributes | Estimate | SE | Df | t-ratio | p-value |
| --- | --- | --- | --- | --- | --- | --- |
| Bacterial OTUs | Weighted.degree | -23.78 | 2.05 | 337 | -11.60 | **<0.001** |
|  | Clustering coefficient | -0.15 | 0.02 | 337 | -8.86 | **<0.001** |
| Fungal OTUs | Weighted.degree | -24.78 | 2.99 | 194 | -8.28 | **<0.001** |
|  | Clustering coefficient | -0.24 | 0.09 | 53 | -2.79 | **0.007** |
| Total OTUs | Weighted.degree | -31.27 | 2.78 | 540 | -11.24 | **<0.001** |
|  | Clustering coefficient | -0.08 | 0.02 | 540 | -4.90 | **<0.001** |

**Table S7**

Pairwise comparisons of network attributes (clustering coefficient and weighted degree) calculated from total OTUs and significantly different OTUs in earthworm gut sections in rape leaves and wheat straw treatments. SE, standard error; Df, degrees of freedom. Significant differences were given in bold (p < 0.05).

| OTUs | Network attributes | Estimate | SE | Df | t-ratio | p-value |
| --- | --- | --- | --- | --- | --- | --- |
| Total OTUs | Weighted.degree | -1.05 | 0.67 | 82 | -1.58 | 0.119 |
|  | Clustering coefficient | -0.02 | 0.07 | 82 | -0.34 | 0.735 |
| Significantly different OTUs | Weighted.degree | -2.70 | 1.16 | 28 | -2.34 | **0.027** |
|  | Clustering coefficient | -0.13 | 0.12 | 28 | -1.09 | 0.284 |


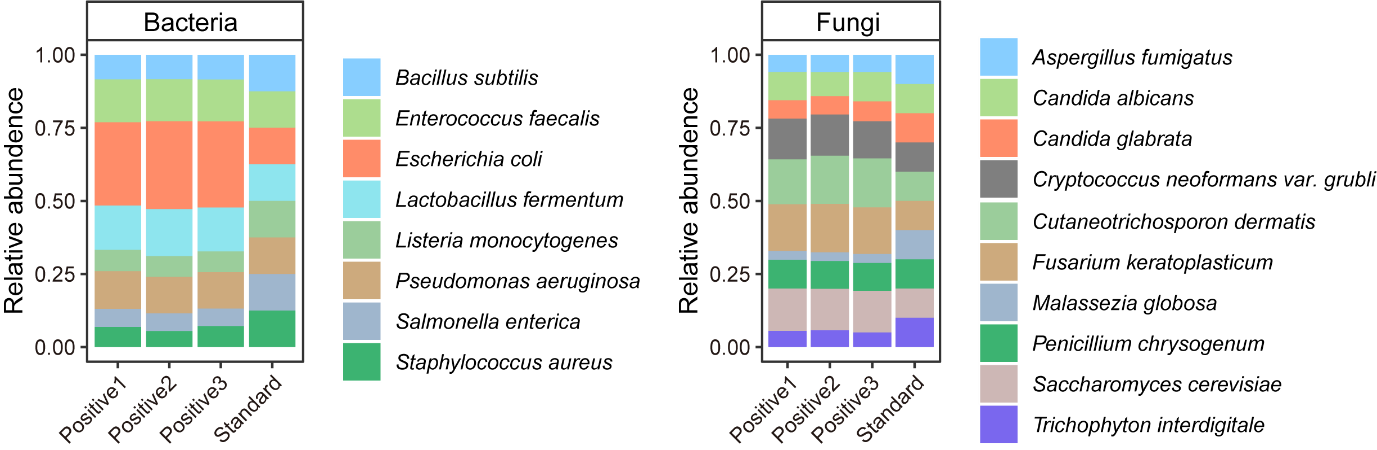


**Fig. S1** Relative abundance of bacterial/fungal species in the positive samples and bacterial (ZymoBIOMICS™ Microbial Community Standard)/ fungal (ZymoBIOMICS™ Microbial Community Standard) standards. The bacterial/fungal communities of positive samples showed concordance between with the suppliers’ specifications. *Escherichia coli* mistakenly classified as *Shigella*. *Shigella* and *Escherichia coli* are very similar that *Shigella* could be classified as a subgenus or a pathovar within *E. coli* rather than a distinct genus.


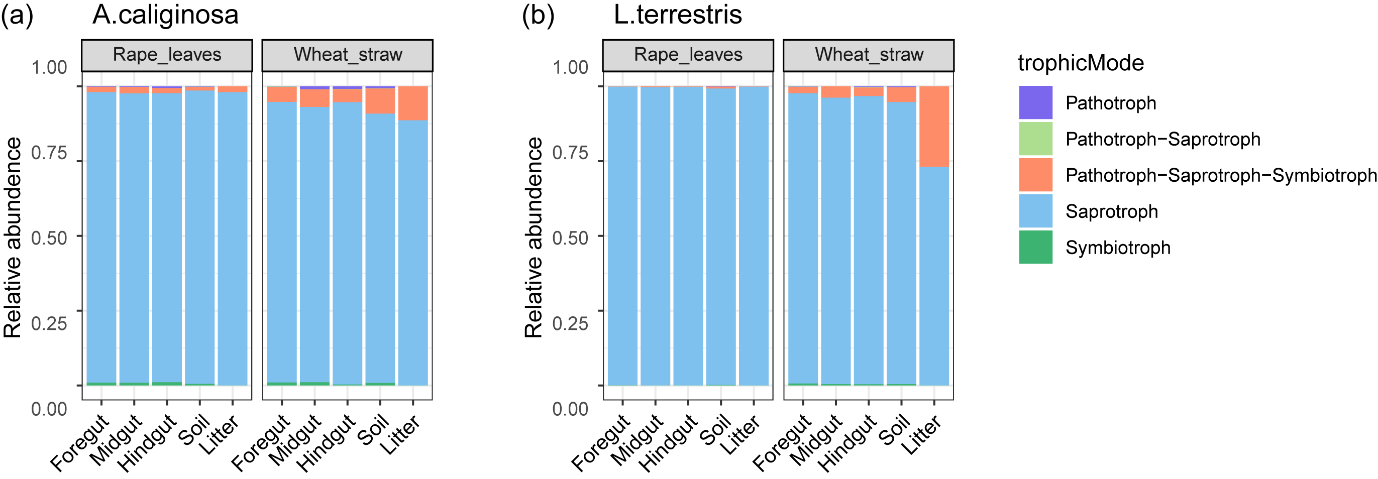


**Fig. S2** Relative abundance (means) of fungal trophic mode in the foregut, midgut and hindgut of *Aporrectodea caliginosa* (a) and *Lumbricus terrestris* (b), as well as in soil and two litter types used as food substrate (rape leaves or wheat straw).


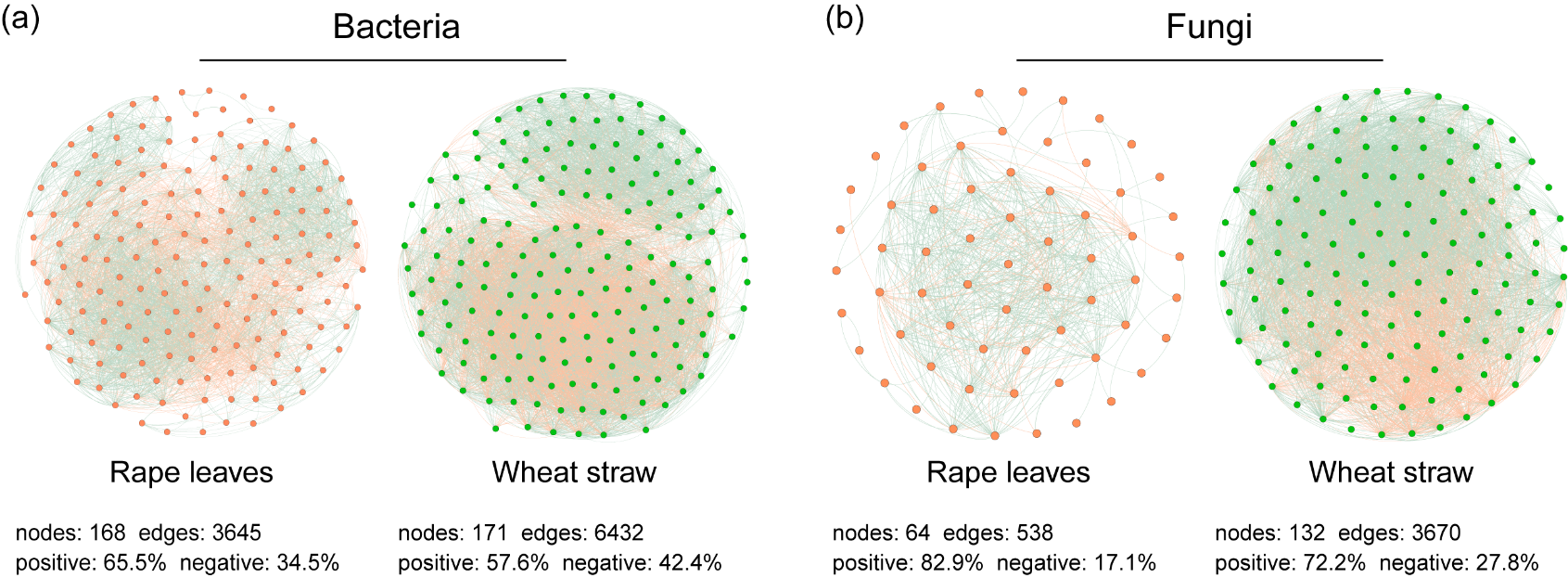


**Fig. S3** Co-occurrence networks based on bacterial (a) and fungal (b) OTUs in the gut of earthworms (pooled for the two species studied, i.e. *Aporrectodea caliginosa* and *Lumbricus terrestris*) fed with different litter materials (rape leaves and wheat straw); orange links, negative relationship; green links, positive relationship; orange nodes, OTUs from rape leaves treatments; green nodes, OTUs from wheat straw treatments.


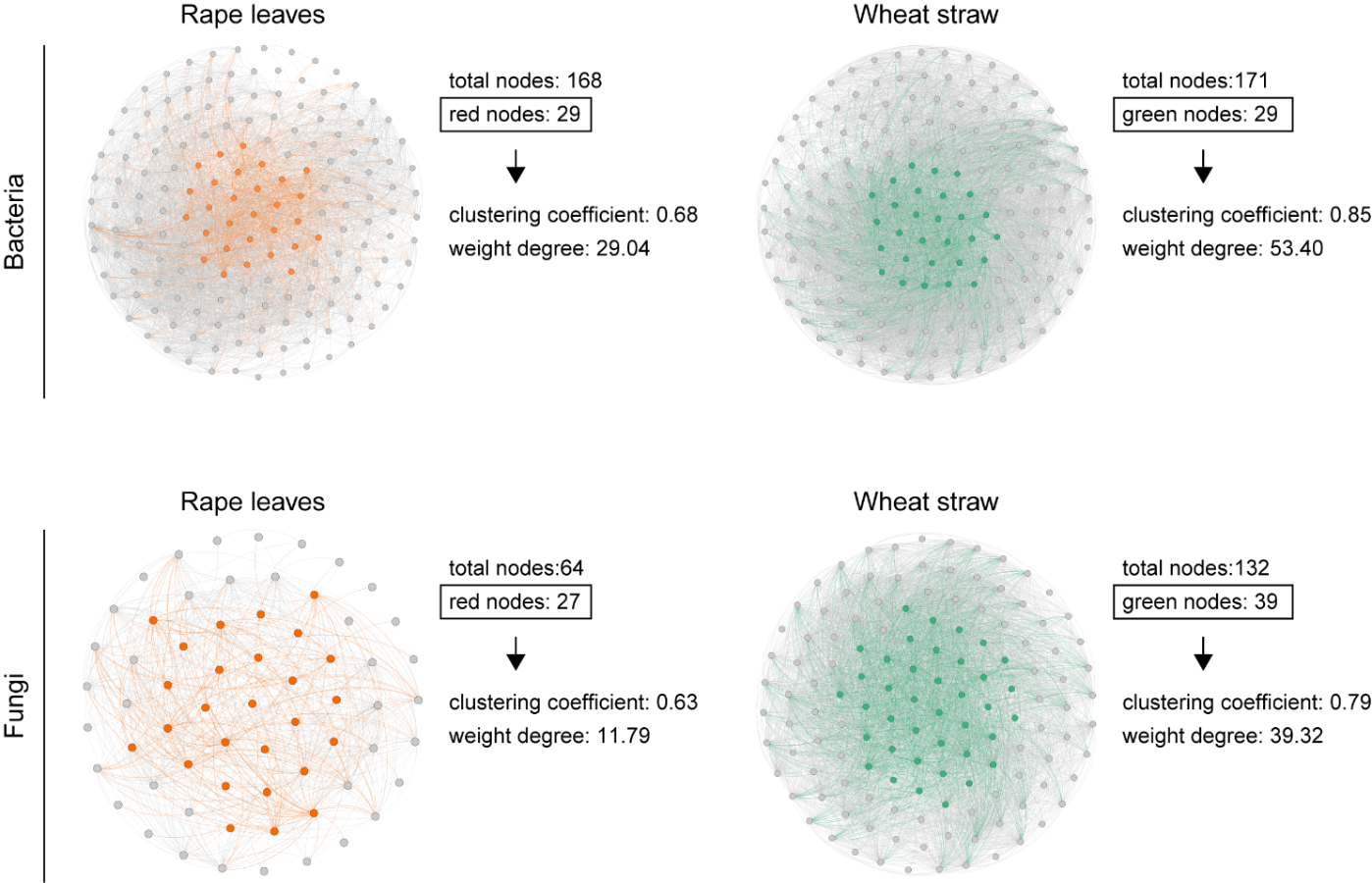


**Fig. S4** Co-occurrence network of significantly different OTUs of bacteria and fungi between rape leaves and wheat straw treatments; orange nodes represent significantly different OTUs from rape leaves treatments, green nodes represent significantly different OTUs from wheat straw treatments, grey nodes represent not significantly different OTUs; the color of each line in the co-occurrence network corresponds to the color of the source node.


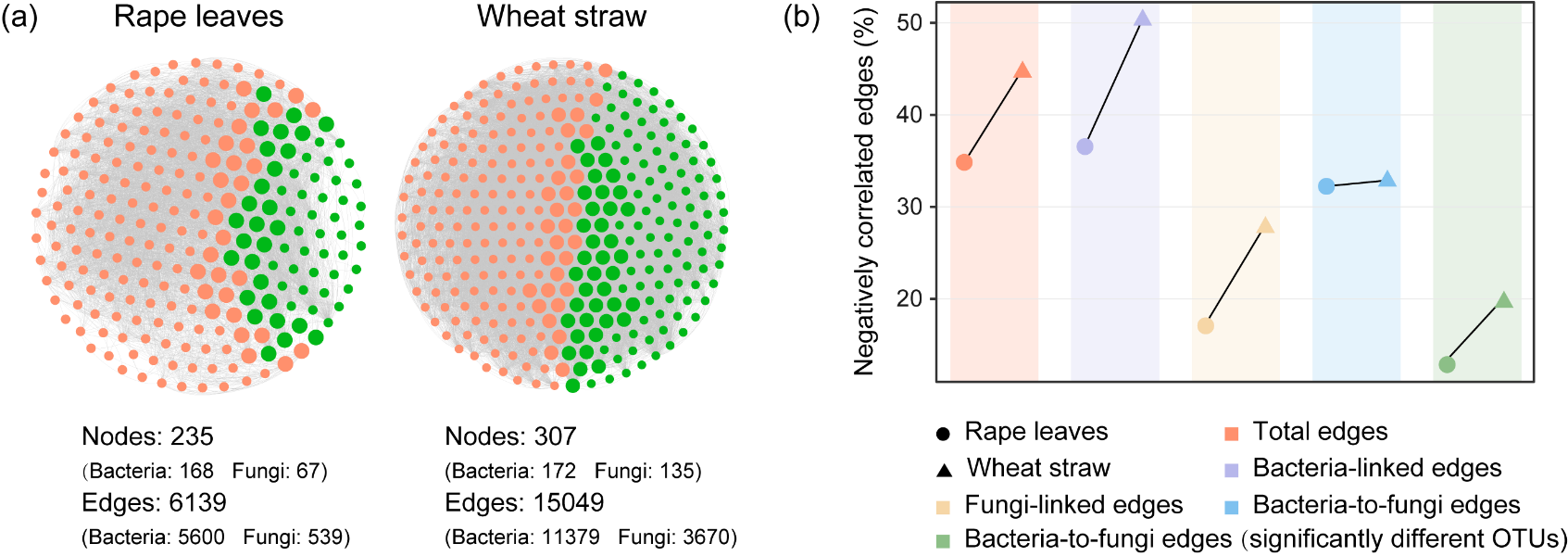


**Fig. S5** (a) Co-occurrence network of combined bacterial and fungal OTUs in the gut of earthworms (pooled for the two species studied, i.e. *Aporrectodea caliginosa* and *Lumbricus terrestris*) fed with rape leaves or wheat straw treatments; orange nodes, bacterial OTUs; green nodes, fungal OTUs; big and small nodes represent significantly different OTUs and not significantly different OTUs, respectively. (b) Percentage of negatively correlated edges in the networks; circles, rape leaves network; triangles, wheat straw network; orange area, all OTUs; purple area, bacterial OTUs; yellow area, fungal OTUs; blue area, connected bacterial and fungal OTUs; green area, significantly different bacterial OTUs and significantly different fungal OTUs.


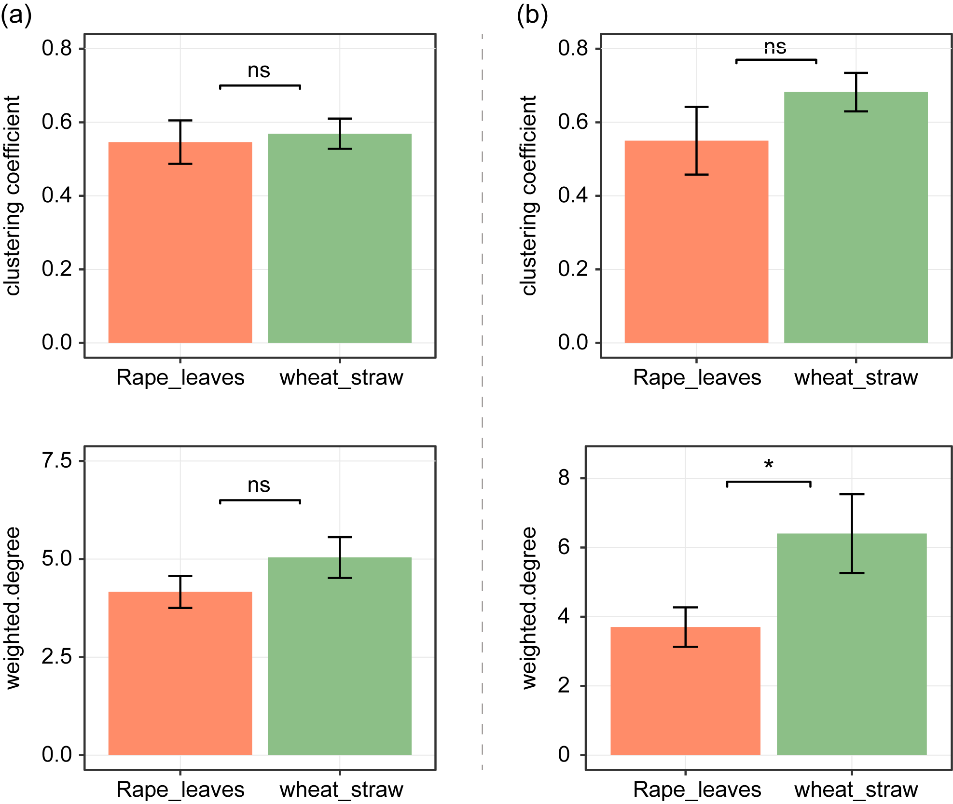


**Fig.S6** Bar plot on the clustering coefficient and weighted degree extracted from networks based on total OTUs (a) and significantly different OTUs (b) in the earthworm gut in rape and wheat straw treatments (means ± SE). Asterisks indicate significant differences, *p < 0.05 (Tukey's HSD test); n.s., not significant.


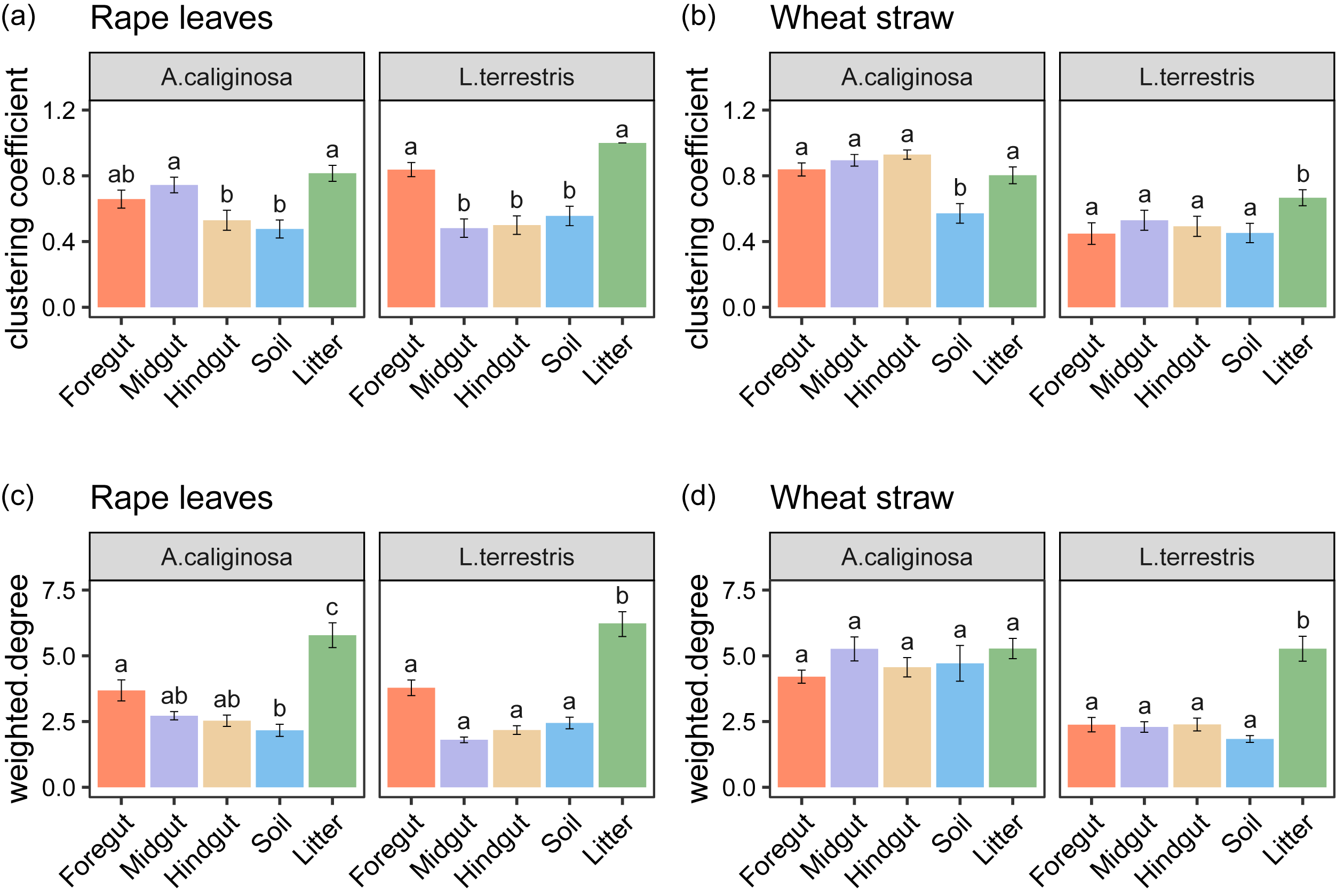


**Fig.S7** Bar plot on the clustering coefficient and weighted degree extracted from networks based on total OTUs in foregut, midgut and hindgut of two earthworm species (*Aporrectodea caliginosa* and *Lumbricus terrestris*), soil and two litter types used as food substrate [rape leaves (a, c) or wheat straw (b, d)] (means ± SE). Different letters indicate significant differences between means (Tukey's HSD test, p < 0.05).

**References**

1. Zhong L, Larsen T, Lu, JZ et al. High litter quality enhances plant energy channelling by soil macro-detritivores and lowers their trophic position. *Ecology* (in press).
2. Sims RW, Gerard BM. Earthworms: Notes for the Identification of British Species. 31st edition. Linnean Society of London and the Estuarine and Coastal Sciences Association, Shrewsbury, UK. 1999
3. Klindworth A, Pruesse E, Schweer T *et al.* Evaluation of general 16S ribosomal RNA gene PCR primers for classical and next-generation sequencing-based diversity studies, *Nucleic Acids Res* 2013;41. https://doi.org/10.1093/nar/gks808.
4. Toju H, Tanabe AS, Yamamoto S *et al.* High-Coverage ITS primers for the DNA-Based identification of Ascomycetes and Basidiomycetes in environmental samples, *PLoS One* 2012;7:e40863. <https://doi.org/10.1371/journal.pone.0040863>.
5. Bolger AM, Lohse M, Usadel B. Trimmomatic: a flexible trimmer for Illumina sequence data, *Bioinformatics* 2014;30:2114-2120. <https://doi.org/10.1093/bioinformatics/btu170>.
6. Lin H, Peddad SD. Multigroup analysis of compositions of microbiomes with covariate adjustments and repeated measures, *Nat Methods* 2024;21:83-91. <https://doi.org/10.1038/s41592-023-02092-7>.
7. Nixon MP, McGovern KC, Letourneau J *et al.* Silverman, scale reliant inference, 2024; <http://arxiv.org/abs/2201.03616>.
8. Fernandes AD, Reid JN, Macklaim JM *et al.* Unifying the analysis of high-throughput sequencing datasets: characterizing RNA-seq, 16S rRNA gene sequencing and selective growth experiments by compositional data analysis, *Microbiome* 2014;2:15. <https://doi.org/10.1186/2049-2618-2-15>.
